# HETEROTOPIC GASTRIC MUCOSA OF THE ESOPHAGUS AS A POTENTIAL CAUSE OF PEPTIC STENOSIS AFTER ROUX-EN-Y GASTRIC BYPASS

**DOI:** 10.1590/0102-6720202400055e1849

**Published:** 2025-01-20

**Authors:** João Victor Vecchi Ferri, Wagner Herbert Sobottka, José Alfredo Sadowski, Gustavo Rodrigues Alves Castro, Vitor Mamoru Haida, Marcela Scardua Cocicov, João Caetano Dallegrave Marchesini

**Affiliations:** 1 São Marcelino Champagnat Hospital – Curitiba (PR), Brazil.

Heterotopic gastric mucosa of the proximal esophagus (HGMPE) is a congenital island of salmon-colored, abnormally located gastric epithelium, commonly present distally to the upper esophageal sphincter (UES). It can vary from microscopic and small foci to extensive and circumferential patches^11^.

Most are largely asymptomatic^8^, found incidentally during esophagogastroduodenoscopies (EGD), with an underestimated prevalence ranging from 2.6 to 21%^1,9^.

However, it can lead to complications such as bleeding, ulceration, neoplastic transformation, acid production, and laryngopharyngeal reflux, specifically chronic cough, throat discomfort, hoarseness, globus sensation, and regurgitation^3^.

Secretion can be acidic, as demonstrated by pH monitoring, and proton pump inhibitor may improve pharyngeal manifestations^7^, but non-acidic mucus can also lead to symptoms^2^.

The inlet patch is commonly a potential site for *Helicobacter pylori* infection. It is closely related to active inflammation and associated with *H. pylori* infection in the stomach^5^. The fact that HGMPE is commonly missed in EGDs can be explained, since it is located in the upper esophagus, a difficult area to examine due to the UES contraction, and is commonly neglected during device removal.

On the other hand, Roux-en-Y gastric bypass (RYGB) is notorious for its potential to treat reflux disease due to various mechanisms: weight loss, reduction of intrabdominal pressure, diversion of bile transit, reduction of acid-producing parietal cells^6,10^. Therefore, addressing patients with presumed gastroesophageal reflux disease (GERD) after this procedure can be challenging. Missed hiatal hernias, intrathoracic migration of the pouch, bulky reservoirs with acid production, short alimentary limbs that allow bile reflux, and gastrogastric fistulas are among other causes.

For example, a 38-year-old female patient presented with dental demineralization and recurrent GERD symptoms in the last 2 years. She was submitted to RYGB 5 years ago at another service due to grade II obesity (body mass index [BMI] 35.9) associated with hypertension and GERD. Her current BMI is 23.8 and presents remission of all diseases. During the investigation, pHmetry revealed increased upper esophageal acid exposure and a DeMeester score of 63; EGD showed an extensive circumferential HGMPE in the upper esophagus, with incipient circumferential ring formation with subtle reduction of the organ's lumen (Figure 1) similar to peptic reaction, with no complications of the pouch (Figure 2). Biopsies found a columnar cardiac epithelium with chronic inflammation; no metaplasia or *H. pylori* were present (Figure 3). She achieved full symptomatic control with dexlansoprazole 60 mg and is being followed to assess the need for interventional therapies. Treatment involves H2 antagonists, proton pump inhibitors, and invasive therapies, such as argon plasma coagulation^2^ and radiofrequency ablation^4^.

**Figure 1:**
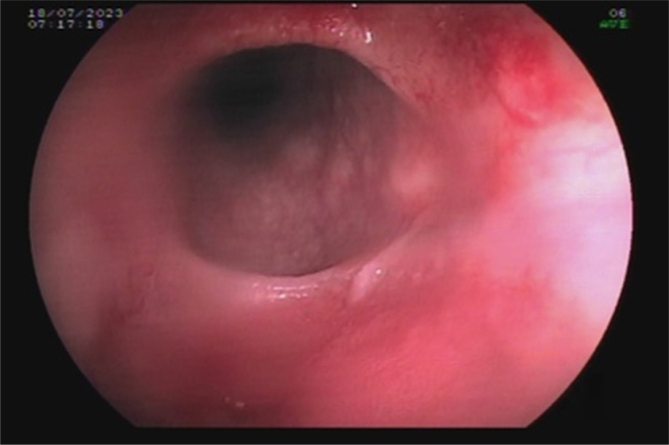
Circumferential ectopic gastric mucosa with distal ring formation, causing subtle reduction of the organ's lumen.

**Figure 2:**
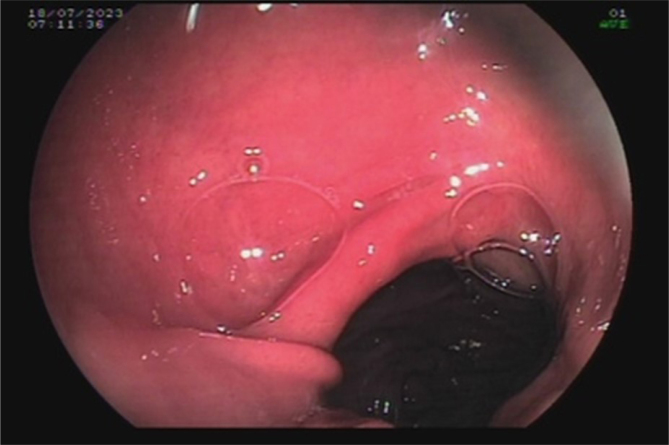
Gastric pouch and gastrojejunal anastomosis.

**Figure 3:**
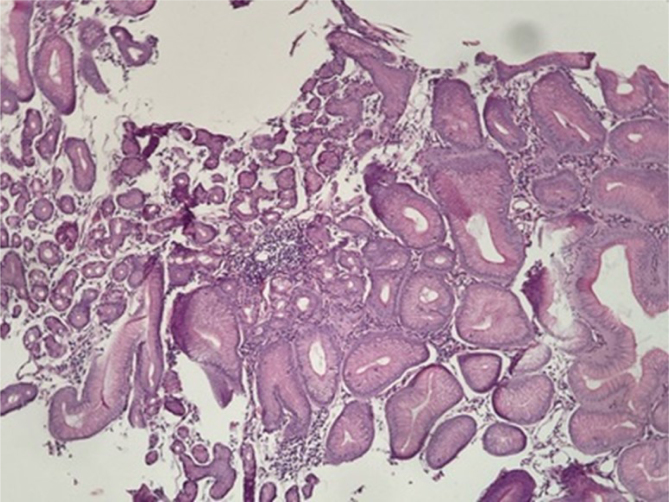
Histopathology revealed columnar cardiac epithelium with chronic inflammation.

Therefore, a detailed esophageal inspection is mandatory in every examination, and it is important to report HGMPE in patients seeking endoscopy due to pharyngolaryngeal symptoms, since it may be the cause. A discrepancy of acid exposure in the upper and lower esophagus should raise suspicion.

## References

[1] Azar C, Jamali F, Tamim H, Abdul-Baki H, Soweid A (2007). Prevalence of endoscopically identified heterotopic gastric mucosa in the proximal esophagus: endoscopist dependent?. J Clin Gastroenterol.

[2] Bajbouj M, Becker V, Eckel F, Miehlke S, Pech O, Prinz C (2009). Argon plasma coagulation of cervical heterotopic gastric mucosa as an alternative treatment for globus sensations. Gastroenterology.

[3] Chong VH, Jalihal A (2010). Heterotopic gastric mucosal patch of the esophagus is associated with higher prevalence of laryngopharyngeal reflux symptoms. Eur Arch Otorhinolaryngol.

[4] Dunn JM, Sui G, Anggiansah A, Wong T (2016). Radiofrequency ablation of symptomatic cervical inlet patch using a through-the-scope device: a pilot study. Gastrointest Endosc.

[5] Gutierrez O, Akamatsu T, Cardona H, Graham DY, El-Zimaity HMT (2003). Helicobacter pylori and hetertopic gastric mucosa in the upper esophagus (the inlet patch). Am J Gastroenterol.

[6] Hawasli A, Phillips A, Tarboush M (2016). Laparoscopic management of reflux after Roux-en-Y gastric bypass using the LINX system and repair of hiatal hernia: a case report. Surg Obes Relat Dis.

[7] Kim EA, Kang DH, Cho HS, Park DK, Kim YK, Park HC (2001). Acid secretion from a heterotopic gastric mucosa in the upper esophagus demonstrated by dual probe 24-hour ambulatory pH monitoring. Korean J Intern Med.

[8] Lupu VV, Ignat A, Paduraru G, Mihaila D, Burlea M, Ciubara A (2015). Heterotopic gastric mucosa in the distal part of esophagus in a teenager: case report. Medicine (Baltimore).

[9] Ohara M (2010). Incidence of heteroptopic gastric mucosa in the upper esophagus in first time narrow banding image endoscopy of consecutive 900 patients. Gastrointest Endosc.

[10] Valezi AC, Campos ACL, Von Bahten LC (2023). Brazilian multi-society position statement on emerging bariatric and metabolic surgical procedures. Arq Bras Cir Dig.

[11] von Rahden BH, Stein HJ, Becker K, Liebermann-Meffert D, Siewert JR (2004). Heterotopic gastric mucosa of the esophagus: literature-review and proposal of a clinicopathologic classification. Am J Gastroenterol.

